# Risk estimation and prediction of the transmission of coronavirus disease-2019 (COVID-19) in the mainland of China excluding Hubei province

**DOI:** 10.1186/s40249-020-00683-6

**Published:** 2020-08-24

**Authors:** Hui Wan, Jing-An Cui, Guo-Jing Yang

**Affiliations:** 1 Jiangsu Key Lab for NSLSCS, School of Mathematical Sciences, Nanjing Normal University, Nanjing, China; 2 Department of Mathematics, School of Science, Beijing University of Civil Engineering and Architecture, Beijing, China; 3 Hainan Medical University, Haikou, 571199 China; 4 Swiss Tropical and Public Health Institute, Socinstrasse 57, Basel, CH-4002 Switzerland; 5 University of Basel, Basel, Switzerland

**Keywords:** COVID-19, Risk estimation and prediction, Intervention measure, Contact tracing, Control reproduction number, Effective daily reproduction ratio, Mathematical model

## Abstract

**Background:**

In December 2019, an outbreak of coronavirus disease (later named as COVID-19) was identified in Wuhan, China and, later on, detected in other parts of China. Our aim is to evaluate the effectiveness of the evolution of interventions and self-protection measures, estimate the risk of partial lifting control measures and predict the epidemic trend of the virus in the mainland of China excluding Hubei province based on the published data and a novel mathematical model.

**Methods:**

A novel COVID-19 transmission dynamic model incorporating the intervention measures implemented in China is proposed. COVID-19 daily data of the mainland of China excluding Hubei province, including the cumulative confirmed cases, the cumulative deaths, newly confirmed cases and the cumulative recovered cases between 20 January and 3 March 2020, were archived from the National Health Commission of China (NHCC). We parameterize the model by using the Markov Chain Monte Carlo (MCMC) method and estimate the control reproduction number (*R*_*c*_), as well as the effective daily reproduction ratio- *R*_*e*_(*t*), of the disease transmission in the mainland of China excluding Hubei province.

**Results:**

The estimation outcomes indicate that *R*_*c*_ is 3.36 (95% *CI*: 3.20–3.64) and *R*_*e*_(*t*) has dropped below 1 since 31 January 2020, which implies that the containment strategies implemented by the Chinese government in the mainland of China are indeed effective and magnificently suppressed COVID-19 transmission. Moreover, our results show that relieving personal protection too early may lead to a prolonged disease transmission period and more people would be infected, and may even cause a second wave of epidemic or outbreaks. By calculating the effective reproduction ratio, we prove that the contact rate should be kept at least less than 30% of the normal level by April, 2020.

**Conclusions:**

To ensure the pandemic ending rapidly, it is necessary to maintain the current integrated restrict interventions and self-protection measures, including travel restriction, quarantine of entry, contact tracing followed by quarantine and isolation and reduction of contact, like wearing masks, keeping social distance, etc. People should be fully aware of the real-time epidemic situation and keep sufficient personal protection until April. If all the above conditions are met, the outbreak is expected to be ended by April in the mainland of China apart from Hubei province.

## Background

Coronaviruses are a group of enveloped viruses with a positive-sense, single-stranded RNA and viral particles resembling a crown, from which the name derives. They belong to the order of *Nidovirales*, family of *Coronaviridae*, and subfamily of *Orthocoronavirinae* [1].

In December 2019, an outbreak of coronavirus disease (later named as COVID-19 by WHO) was identified in Wuhan, China and, later on, detected in other parts of China. By 27 February, the new virus had infected 78 824 people and killed 2788 people in China [2]. Besides China, more than 4440 people had been infected and 67 died in at least 48 countries and regions [3]. Currently, there are no vaccines or anti-viral treatments officially approved for the prevention or management of the diseases. The outbreaks are still on-going [3].

The basic reproduction number *R*_0_ is the average number of secondary infections due to an infective case during the infectious period when everyone else in the population is susceptible [4]. While the basic reproduction number with control measures is defined as the control reproduction number *R*_*c*_. At the early stage of the outbreak, estimation of *R*_0_/ *R*_*c*_ is crucial for determining the potential and severity of an outbreak, and providing precise information for designing and implementing disease outbreak responses, namely the identification of the most appropriate, evidence-based interventions, mitigation measures and the determination of the intensity of such programs in order to achieve the maximal protection of the population with the minimal interruption of social-economic activities [5, 6].

Recently, some papers have been released as pre-prints or undergone peer-review and published to estimate *R*_0_ and the risk of outbreak. Li et al. [7] analyzed data on the first 425 confirmed cases in Wuhan and determined the epidemiologic characteristics of COVID-19. Based on their estimates, the mean incubation period was 5.2 days, and *R*_0_ was 2.2, which is in line with the result estimated by Riou et al. [8]. Zhao et al. [9] assessed the unreported number of COVID-19 cases in China in the first half of January with the estimation of *R*_0_ 2.56. Considering the impact of the variations in disease reporting rate, Zhao et al. [10] modelled the epidemic curve of COVID-19 cases, in the mainland of China from 10 January to 24 January 2020, through the exponential growth and concluded that the mean *R*_0_ ranged from 2.24 to 3.58 associated with 2-fold to 8-fold increase in the reporting rate. Li et al. [11] conducted a mathematical modeling study using five independent methods to assess *R*_0_ of COVID-19. Their results illustrated that *R*_0_ dropped from 4.38 to 3.41 right after the lockdown of Wuhan city. Over the epidemic period of the study COVID-19 had an average *R*_0_ of 3.39. Moreover, Tang et al. formulated a deterministic compartmental model. Their estimations based on likelihood and dynamical model analysis showed that *R*_0_ with control measures might be as high as 6.47 [6]. Most recently, Chen et al. [12] developed a Bats-Hosts-Reservoir-People transmission network model to simulate the potential transmission from the infectious sources to human. The estimated values of *R*_0_ were 2.30 from reservoir to person and 3.58 from person to person. We noticed that the estimations of *R*_0_ in varied studies are different. As mentioned in references of [6, 13], variability in the estimation of the basic reproduction number is a general recognized methodological issue, and standardized methods both for calculating and reporting *R*_0_ are still missing. Furthermore, the value of *R*_0_ may vary with key clinical parameters inferred from data which depend on the time period, quality, accuracy, and reliability. To better quantify the evolution of the interventions, Tang et al. fitted the previously proposed model in reference [6] to the data available until 29 January 2020 and re-estimated the effective daily reproduction [14]. There are also some literatures focusing on the prediction of COVID-19 development trend. Wang et al. formulated a complex network model and analyzed the possible time node and the risk impact of resumption on secondary outbreak in Wuhan and surrounding areas [15]. Roosa et al. [16] utilized several dynamic models to forecast the cumulative number of confirmed cases in the coming 5, 10, and 15 days in Hubei province, and the overall trajectory of the epidemic in China excluding Hubei.

With the gradual alleviation of the epidemic situation in the mainland of China excluding Hubei province, considering the pressure of economic operation and the needs of people’s normal production and life, some places have adjusted the primary response of epidemic prevention and control to the secondary response [17]. In this situation, some critical questions need to be answered promptly. Does the reduction of the emergency response level mean that people can fully or partially relieve from self-production? When can people return back to normal life?

The aim of this study is to evaluate the effectiveness of varied interventions and self-protection measures, estimate the risk of partial lifting control measures and predict the epidemic trend of the virus in the mainland of China excluding Hubei province by establishing a COVID-19 transmission model incorporating the intervention measures implemented and fitting the data obtained from the National Health Commission of China (NHCC).

## Methods

### Establishment of COVID-19 transmission dynamic model

Based on the clinical progression of the disease, epidemiological status of the individuals and intervention measures (including travel restriction, body temperature measurement, close contact tracing, self-isolation and protection, etc.), we propose a novel deterministic COVID-19 transmission model. We parameterize the model using data of the mainland of China excluding Hubei province obtained from NHCC, and estimate the control reproduction number as well as the effective daily reproduction ratio of the disease transmission.

The population was grouped into various compartments, namely susceptible (S), exposed (E), infectious with symptoms (I), infectious but asymptomatic (A), isolated susceptible (Si), quarantined infected pending for confirmation (Q), hospitalized (H), and recovered (R). We assume that recovered individuals have immunity during the rest epidemic period. Let *N*(*t*)=*S*(*t*)+*E*(*t*)+*I*(*t*)+*A*(*t*)+*R*(*t*) be the total number of individuals in the free community. In order to fit the data, we explicitly generated additional two groups, i.e. the cumulative number of recovered *R*_*h*_(*t*) and dead cases *D*(*t*) from hospital. The total number of cumulative reported cases is set to be *T*(*t*). All the state variables are summarized in Table 1.

**Table 1:** State variables and initial values in Model (1)

Definitions	State variables	Initial values	Geweke	Source
Susceptible population	*S*(*t*)	1 336 210 000	–	[27]
Exposed population	*E*(*t*)	501.23	0.92956	MCMC
Symptomatic infected population	*I*(*t*)	0.22839	0.95511	MCMC
Asymptomatic infected population	*A*(*t*)	991.29	0.81969	MCMC
Isolated susceptible population	*S*_*i*_(*t*)	0	–	Data
Quarantined infected population	*Q*(*t*)	0	–	Data
Infected population in hospitals	*H*(*t*)	21	–	Data
Recovered population	*R*(*t*)	240.76	0.63851	MCMC
Recovered population from hospitals	*R*_*h*_(*t*)	0	–	Data
Cumulative number of dead cases in hospitals	*D*(*t*)	0	–	Data
Total number of reported cases	*T*(*t*)	21	–	Data

*MCMC* Markov Chain Monte Carlo method; *Data* COVID-19 daily data excluding Hubei province archived from NHCC

Due to the travel restriction, migrations from/to Hubei province and other regions are ignored. Birth and natural death are also neglected. With the increase of the cumulative number of confirmed cases, the probability of contact transmission among the informed susceptible populations would certainly reduce ([18–20], etc.). To better quantify the varied interventions and self-protection measures, we assume the contact rate to be time-dependent *c*(*t*)=*q*_1_(*t*)*c*_0_, where *c*_0_ is the initial contact rate and *q*_1_(*t*) is the intervention coefficient with respect to contact. Here we assume that *q*_1_(*t*)=*e*^−*δT*(*t*)^, which is dependent on the total number of cumulative confirmed cases *T*(*t*) and is monotone decreasing with *T*(*t*), so as to well reflect the impact of media coverage on people’s psychology and behaviors. *c*(*t*)=*c*_0_ for *T*=0 and $\lim \limits _{t \rightarrow \infty } c(T)=0.$ It should be mentioned that the contact function *c*(*t*) in [14] is also assumed to be time-dependent, but it is not dependent on state variables. Let the transmission probability be *β*. Thus, the incidence rate can be given by $\beta c(t)\left (I+ \xi A\right)\frac {S}{N},$ where *ξ* is the correction factor of transmission probability with asymptomatic infectious individuals.

By frequent monitoring of body temperature and diagnoses in hospitals, symptomatic infectious individuals can be detected. The detection rate is assumed to be *q*_2_*I*(*t*). Infected individuals in *Q* class can be confirmed at the rate of *ηQ*(*t*) by nucleic acid testing. Additionally, close contact tracing followed by quarantine and isolation is a critical control measure. We assume that, once a case is confirmed, *q*_3_ individuals would be traced. Therefore, *q*_3_(*q*_2_*I*(*t*)+*ηQ*(*t*)) individuals would be traced in a unit time, which is dependent on the number of new confirmed cases *q*_2_*I*(*t*)+*ηQ*(*t*). We also assume that, among these traced individuals, $\frac {S}{N}$ fraction parts are susceptible, $\frac {E}{N}$ fraction parts are exposed, $\frac {I}{N}$ fraction parts are infectious with symptoms and $\frac {A}{N}$ fraction parts are infectious but asymptomatic. $\frac {R}{N}$ fraction parts are recovered, which are not needed to be isolated due to protective immunity and will remain in the *R* class until the end of the epidemic.

**Fig. 1:**
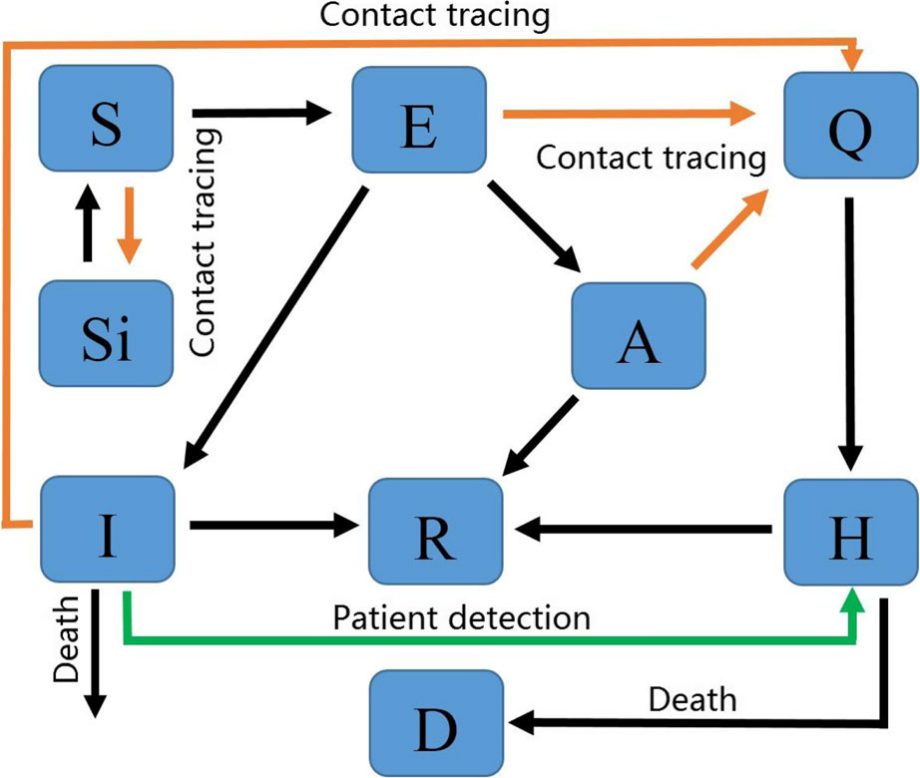
Flow chart of COVID-19 with intervention measures

The disease transmission flow chart is depicted in Fig. 1 and other parameters are summarized in Table 2. Based on the above assumptions, we formulate the following model to describe the transmission dynamics of COVID-19.

1 $$ {}\left\{\begin{array}{ll} \frac {dS(t)}{dt}&=-\beta c(t)\left(I+ \xi A\right)\frac{S}{N}-q_{3}(q_{2} I+\eta Q)\frac{S}{N}+\mu S_{i},\\ \frac{dE(t)}{dt}&=\beta c(t)\left(I+\xi A\right)\frac{S}{N}-\phi E-q_{3}\left(q_{2} I+\eta Q\right)\frac{E}{N},\\ \frac{dI(t)}{dt}&=\theta \phi E-\gamma_{I} I-dI-q_{2} I-q_{3}\left(q_{2} I+\eta Q\right)\frac{I}{N},\\ \frac{dA(t)}{dt}&=(1-\theta)\phi E-\gamma_{A} A-q_{3}\left(q_{2} I+\eta Q\right)\frac{A}{N},\\ \frac{dS_{i}(t)}{dt}&=q_{3}\left(q_{2} I+\eta Q\right)\frac{S}{N}-\mu S_{i},\\ \frac{dQ(t)}{dt}&=q_{3}\left(q_{2} I+\eta Q\right)\frac{E+I+A}{N}-\eta Q,\\ \frac{dH(t)}{dt}&=q_{2} I+\eta Q-dH-\gamma_{H}H,\\ \frac{dR(t)}{dt}&=\gamma_{I} I+\gamma_{A} A+\gamma_{H}H,\\ \frac{dR_{h}(t)}{dt}&=\gamma_{H}H,\\ \frac{dD(t)}{dt}&=dH.\\ \frac{dT(t)}{dt}&=q_{2} I+\eta Q.\\ \end{array}\right.  $$

**Table 2:** Definition of the parameters

Interpretations	Parameters	Values	Geweke	Source
Probability of transmission per contact	*β*	0.054043	0.92594	MCMC
Initial contact rate	*c*_0_	40.319	0.99203	MCMC
Transition rate of exposed individuals	*ϕ*	1/5.2	–	[7]
to the infected class				
Probability of having symptoms	*θ*	0.6628	0.91799	MCMC
among infected individuals				
Transition rate of quarantined infected	*η*	17.379	0.59129	MCMC
individuals to hospital class				
Recovery rate of symptomatic	*γ*_*I*_	0.15796	0.7351	MCMC
infectious individuals				
Recovery rate of asymptomatic	*γ*_*A*_	0.55671	0.99168	MCMC
infected individuals				
Recovery rate of quarantined	*γ*_*H*_	0.035352	0.97782	MCMC
infected individuals				
Disease-induced death rate	*d*	5.5888×10^−4^	0.98847	MCMC
Correction factor of transmission probability	*ξ*	0.80987	0.74505	MCMC
with asymptomatic infectious individuals				
Rate at which the quarantined uninfected	*μ*	1/14	–	[2]
contacts were released				
Intervention coefficient with respect	*q*_1_	–	–	Assumption
to contact				
Intervention parameter with respect	*q*_2_	0.47218	0.9302	MCMC
to patient detection				
Intervention parameter with respect	*q*_3_	2.6954	0.55476	MCMC
to close contact tracing				
Exponential decreasing rate of contact rate	*δ*	2.8286×10^−4^	0.99962	MCMC

*MCMC* Markov Chain Monte Carlo method

According to the concept of next generation matrix in reference [21] and the basic reproduction number presented in reference [22], we calculate the basic reproduction number with control measures, i.e. the control reproduction number, *R*_*c*_, of COVID-19, which is given by

$$R_{c}=\frac{\beta \theta c_{0}}{\gamma_{I}+d+q_{2}}+\frac{\beta \xi(1-\theta)c_{0}}{\gamma_{A}}.$$

With the spreading of the COVID-19, intensive intervention measures have been implemented increasingly and people gradually enhanced self-protection. In order to quantity the daily reproduction number, inspired by Tang et al. [14], the initial contact rate *c*_0_ in the formula of *R*_*c*_ is replaced by the aforementioned time-dependent contact rate *c*(*t*) to reflect the changes of intervention measures and people’s behaviors. Thereby, we define

$$R_{e}(t)=\frac{\beta \theta c_{0}e^{-\delta T(t)}}{\gamma_{I}+d+q_{2}}+\frac{\beta \xi(1-\theta)c_{0}e^{-\delta T(t)}}{\gamma_{A}}$$

as the effective daily reproduction ratio, the average number of new infections induced by a single infected individual during the infectious period at time *t*. The basic reproduction number or the control reproduction number, which is not time-dependent, can depict the transmission risk in the early phase of disease transmission. While the time-dependent effective daily reproduction ratio can evaluate the transmission risk changing over time.

### Data source

**Fig. 2:**
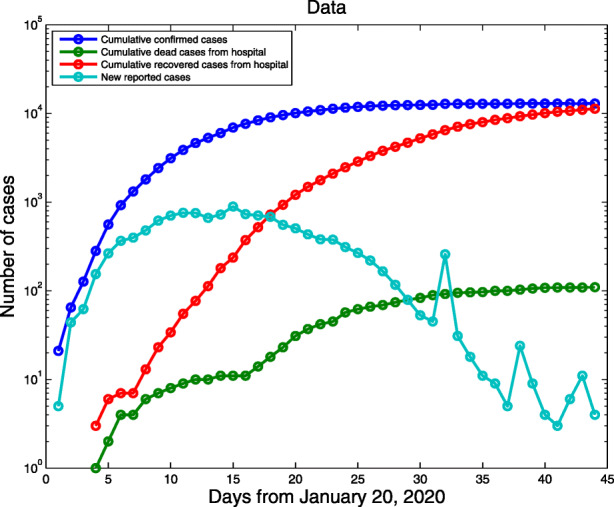
Reported data of the mainland of China excluding Hubei

COVID-19 daily data excluding Hubei province were archived from NHCC from 20 January (the first day of confirmed cases reported) to 3 March 2020 (Fig. 2) [2]. The data include the cumulative confirmed cases, the cumulative number of deaths, newly confirmed cases and the cumulative number of recovered cases. The data from 20 January to 24 February were fitted to parameterize the model and the data from 25 February to 3 March were used for comparison of the predicted curves and real data.

### Parameter estimation

We used the Markov Chain Monte Carlo (MCMC) method with an adaptive Metropolis-Hastings (M-H) algorithm to fit the model. The M-H algorithm, a powerful Markov chain method to simulate multivariate distributions, was executed by using the MCMC toolbox introduced in reference [23]. This toolbox provides tools to generate and analyze Metropolis-Hastings MCMC chains using multivariate Gaussian proposal distribution. The covariance matrix of the proposal distribution can be adapted during the simulation according to adaptive schemes described in references of [24–26].

**Fig. 3:**
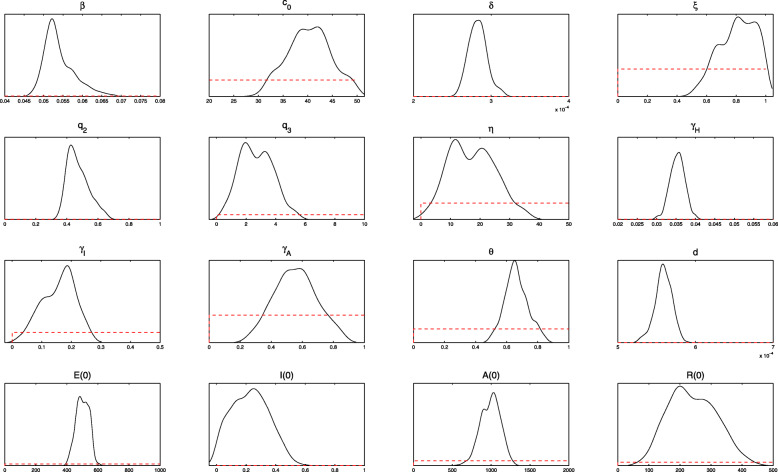
Posterior distribution (solid line) and prior distribution (dash line) of each parameter. x-axis is the parameter value, y-axis is the density

The algorithm is run for 110 000 iterations with a burn-in of the first 80 000 iterations, and the Geweke convergence diagnostic method is employed to assess the convergence of chains. At the significance level of 5% (the critical value of *z* is 1.96), all parameters and initial values estimated do not reject the original hypothesis of convergence to a posterior distribution (Fig. 3).

### Simulations

The population of the mainland of China excluding Hubei province is around 1 336 210 000 [27], which can be set to be the value of *S*(0). 21 confirmed cases were reported on 20 January 2020 for the first time and the numbers of recovered and dead individuals were both 0. It can be assumed that nobody has been traced at the beginning. Therefore, we set *S*_*i*_(0)=*Q*(0)=*R*_*h*_(0)=*D*(0)=0, and *H*(0)=*T*(0)=21. The quarantined susceptible individuals were isolated for 14 days, thus *μ*=1/14 [2]. According to reference [7], the incubation period of COVID-19 is about 5.2 days, thus *ϕ* can be set to be 1/5.2.

## Results

**Fig. 4:**
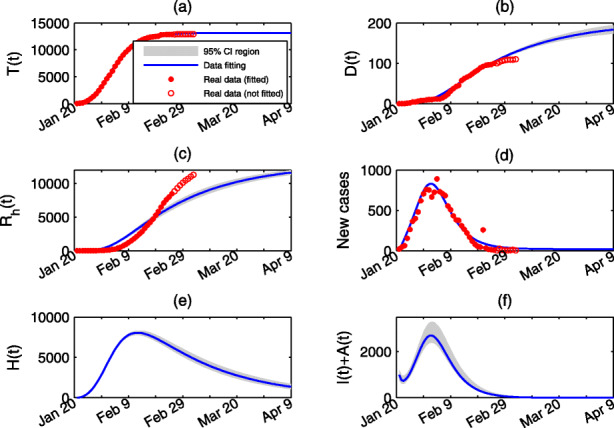
Data fitting and prediction. The abscissa axis is the time. The shaded area is the 95% confidence interval region; The blue solid line is the result of the date fitting; The red stars are the real data. **a** Cumulative confirmed cases; **b** Cumulative dead cases from hospitals; **c** Cumulative recovered cases; **d** The number of new cases; **e** The number of hospitalized individuals; **f** The number of infectious individuals

It can be seen that our model yields a relatively good visual fit to the epidemic curves (see Fig. 4). The MCMC estimation results of each parameters and initial values of some state variables are given in Tables 1 and 2. By fitting the data, we estimated the control reproduction number *R*_*c*_ to be 3.36 (95% *CI*: 3.20–3.64).

**Fig. 5:**
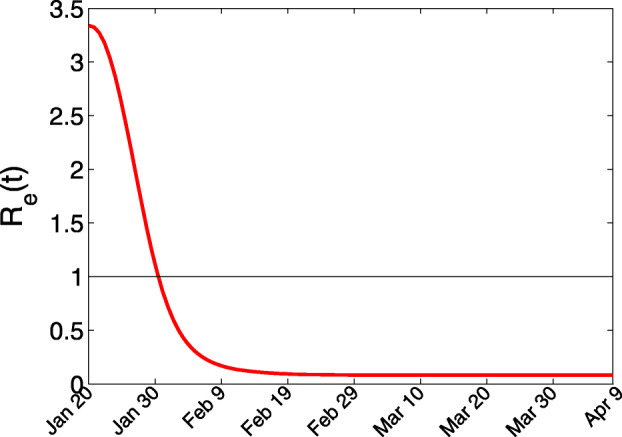
Effective reproduction number

Using the estimated parameter values and the number of cumulative cases *T*(*t*), the effective daily reproduction ratio *R*_*e*_(*t*) can be calculated (Fig. 5). The result demonstrates that *R*_*e*_(*t*) has dropped sharply from 3.34 (*R*_*e*_(1)=3.34) on 20 January to 0.89 (*R*_*e*_(12)=0.89, less than 1) on 31 January 2020, which implies that the integrated control strategies implemented in the mainland of China excluding Hubei has successfully reduced transmission intensity and prevented the epidemic growth in a short time frame.

Under the current rigorous integrated control and self-protection measures, the time series of *T*(*t*) depicted in Fig. 4a shows that the cumulative number of confirmed cases will continue growing slowly for some duration and tend to its predicted maximum, which is 13 155. Besides, although the number of hospitalized individuals has peaked on around 12 February 2020, but it will not shrink to zero in the near future (Fig. 4e). Obviously, once people start relieving self-isolation and protection, new infections would occur given the existence of undetected infectious individuals. It is the number of undetected infectious individuals that determines when people’s lives are able to return back to normal. Hence we should closely follow the total number of *I*(*t*) and *R*(*t*). Fig. 4f displays that the number of infectious individuals has been decreasing gradually since the end of January. However, it will not descent down to 1 until late March, which infers that people should be fully aware of the real-time epidemic situation and keep personal protect before April.

Using the estimated parameter values and the expression of the effective reproduction ratio *R*_*e*_(*t*), the threshold value of the intervention coefficient with respect to contact, *q*_1_, can be calculated, which is 0.3. This implies that in order to block the continuous spread of the virus, the value of *q*_1_ must be less than 0.3 to guarantee *R*_*e*_(*t*) is below 1. In other words, the contact rate should be kept below 30% of the normal level.

**Fig. 6:**
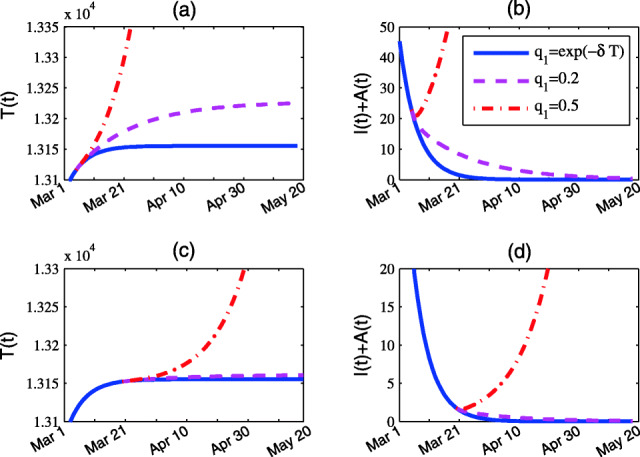
Comparison of scenarios with different contact rates. The abscissa axis is the time. The blue solid line is the case with no changes (*q*_1_=*exp*(−*δT*(*t*))); The purple dash line is the case with *q*_1_=0.2; The red dash-dot line is the case with *q*_1_=0.5. **a**–**b** Adjust the contact rate from 5 March 2020; **c**–**d** Adjust the contact rate from 20 March 2020

To examine the impact of partial lifting control measures and personal protection, we plot the predicted time series of the number of cumulative confirmed cases, *T*(*t*), and the number of the infectious individuals, *I*(*t*)+*A*(*t*), with different contact rates (Fig. 6). Assuming that the adjusting time is from 5 March 2020, Figs. 6a and b illustrate that contact rate with 20% of the initial value *c*_0_ will not cause the disease re-bounce. However, the epidemic period will be extended for about 40 days until early May and the cumulative number of confirmed cases will increase by around 0.5*%* to 13 227. While if the starting time of the adjusting is postponed to 20 March, the epidemic time of disease will be extended for about one week and the cumulative number of confirmed cases will increase by only around 0.05*%* to 13 161, compared with the scenario of no changes. Nevertheless, if the contact rate is half of the initial value, i.e. *q*_1_=0.5 and *R*_*e*_(*t*)=1.68, COVID-19 will re-bounce on a large scale in a short time frame, even if the starting time is postponed to 20 March. The corresponding analysis results are also listed in Table 3 for comparison.

**Table 3:** The impact of partial lifting control measures and personal protection

Starting time of adjustment	Maximum of cumulative confirmed cases	Epidemic period
No adjustment (*c*(*t*)=*c*_0_*e*^−*δT*(*t*)^)	13 155	70 days
5 March (*c*(*t*)=0.2*c*_0_)	13 227	110 days
5 March (*c*(*t*)=0.5*c*_0_)	More than 447 million	More than 365 days
20 March (*c*(*t*)=0.2*c*_0_)	13 161	77 days
20 March (*c*(*t*)=0.5*c*_0_)	More than 445 million	More than 365 days

## Discussion

In this paper, we proposed a novel COVID-19 transmission model incorporating the intervention measures implemented in China. Particularly, we adopted two novel function forms which dynamically captured the real-time endemic situation. The first one is the contact rate, *c*(*t*), which was assumed to be dependent on the cumulative number of confirmed cases to better quantify the varied interventions and self-protection measures. The other one is the contact tracing function, which was dependent on the number of new cases.

We evaluated the impact of partial lifting control measures on COVID-19 transmission. Our results show that relaxing self-protection too early may lead to a prolonged transmission period and more people would be infected, and may even cause the second wave of epidemic or outbreaks. The reduction of the emergency response level does not mean that people can be relaxed thoroughly. At least until the end of March, life will not be able to return to normal.

In the process of recovery of production and life, we should pay attention to take protective measures to minimize the contact between people, such as wearing masks, keeping social distance, and avoiding crowded places to cut the risk of infection. By calculating the effective reproduction ratio, we assert that people’s contact rate should be kept below 30% of the normal level and the lower the better.

Our study outcomes are based on the assumption of no imported cases from Hubei and other regions. However, new endemic foci outside China are formed, such as Republic of Korea, Italy, Iran, Japan, etc. The WHO recently upgraded the global risk of the epidemic to ‘very high’. Although the current endemic situation in China is under control, these newly formed epidemic foci could generate the risk of second outbreak wave due to imported cases via population migration cross border. With the development of the critical epidemic situation in other countries, it is very important to maintain and strengthen the quarantine of entry personnel. The impact of international mobility on the transmission of COVID-19 will be studied in our future works.

We concentrated on the epidemic situation in the mainland of China excluding Hubei province is due to the significant differences of transmission dynamics between Wuhan, Hubei, and the rest of the country. In Wuhan, because of the sudden appearance of the disease, it took much longer time to recognize and understand the disease transmission than other regions. It requires certain time from unknown to known. Therefore, the data accuracy in Wuhan, Hubei is a major issue for parameters’ calibration. In addition, the sudden large outbreak in Wuhan, Hubei exhausted all medical resources in a short time. A more targeted model considering medical resource capacity will be anticipated in the future.

Our results manifest that the model yields a good visual fit to the epidemic curve except for the cumulative recovered cases. The number of predicted recovered cases is less than the number of actual recovered individuals in the later period of data fitting. The possible reason is that the recovery rate of hospitalized individuals was increasing with the improvement of treatment level and the increase of medical resources. In addition, due to increased contact tracing efficiency, large quantity of mild symptomatic cases were detected and hospitalised which has higher recover rate than other classified cases. A time-dependent recovery rate may be more suitable to fit the real data. Furthermore, age is a key parameter determining the mortality and recover rates. It is ideal to set up age-structured agent-based or meta population-based deterministic models which can capture this important risk factor. In this work, the initial value of the susceptible class *S*(0) was set to be the population of the mainland of China excluding Hubei province. Actually, the number of susceptible individuals is not as many as this number considering the very strict control measures implemented and the difference of behaviors between different age groups. A proper method to estimate the accurate number of susceptible individuals should be studied in the future. Besides, the impact of limited medical resources is another important issue that is not incorporated in our model. The transmission dynamics of COVID-19 accounting for the potential negative effects of health systems being overwhelmed on mortality will be studied in our future work.

## Conclusions

In conclusion, to ensure the COVID-19 pandemic ending rapidly, it is necessary to maintain the current integrated control intervention and self-protection measures, including travel restriction, quarantine of entry, contact tracing followed by quarantine and isolation and reduction of contact, like wearing masks, keeping social distance, etc. People should be fully aware of the real-time epidemic situation and keep sufficient personal protection until April. If all the above conditions are met, the outbreak is expected to be ended by April in the mainland of China apart from Hubei province.

## Data Availability

Not applicable.

## Abbreviations

COVID-19: Corona Virus Disease 2019
NHCC: National Health Commission of the People’s Republic of China
MCMC: Markov Chain Monte Carlo method
*R*_0_: Basic reproduction number
*R*_*c*_; Control reproduction number; *R*_*e*_(*t*): Effective daily reproduction ratio
MCMC: Markov Chain Monte Carlo method.
